# Calculated relative biological effectiveness (RBE) for initial DNA double-strand breaks (DSB) from flattening filter and flattening filter-free 6 MV X-ray fields

**DOI:** 10.1259/bjro.20200072

**Published:** 2021-07-05

**Authors:** Hisashi Nakano, Daisuke Kawahara, Satoshi Tanabe, Satoru Utsunomiya, Takeshi Takizawa, Madoka Sakai, Toshimichi Nakano, Atsushi Ohta, Motoki Kaidu, Hiroyuki Ishikawa

**Affiliations:** 1 Department of Radiation Oncology, Niigata University Medical and Dental Hospital, 1-757 Asahimachi-dori, Chuo-ku, Niigata, Japan; 2 Department of Radiation Oncology, Institute of Biomedical & Health Sciences, Hiroshima University, 1-2-3 Kasumi, Minami-ku, Hiroshima-shi, Hiroshima, Japan; 3 Department of Radiological Technology, Niigata University Graduate School of Health Sciences, 2-746 Asahimachi-dori, Chuo-ku, Niigata, Japan; 4 Department of Radiology and Radiation Oncology, Niigata University Graduate School of Medical and Dental Sciences, 1-757 Asahimachi-dori, Chuo-ku, Niigata, Japan; 5 Department of Radiation Oncology, Niigata Neurosurgical Hospital, Niigata, 3057 Yamada, Nishi-ku, Niigata, Japan

## Abstract

**Objectives:**

We evaluated the radiobiological effectiveness based on the yields of DNA double-strand breaks (DSBs) of field induction with flattening filter (FF) and FF-free (FFF) photon beams.

**Methods:**

We used the particle and heavy ion transport system (PHITS) and a water equivalent phantom (30 × 30 × 30 cm^3^) to calculate the physical qualities of the dose-mean lineal energy (y_D_) with 6 MV FF and FFF. The relative biological effectiveness based on the yields of DNA-DSBs (RBE_DSB_) was calculated for standard radiation such as 220 kVp X-rays by using the estimating yields of SSBs and DSBs. The measurement points used to calculate the in-field y_D_ and RBE_DSB_ were located at a depth of 3, 5, and 10 cm in the water equivalent phantom on the central axis. Measurement points at 6, 8, and 10 cm in the lateral direction of each of the three depths from the central axis were set to calculate the out-of-field y_D_ and RBE_DSB_.

**Results:**

The RBE_DSB_ of FFF in-field was 1.7% higher than FF at each measurement depth. The RBE_DSB_ of FFF out-of-field was 1.9 to 6.4% higher than FF at each depth measurement point. As the distance to out-of-field increased, the RBE_DSB_ of FFF rose higher than those of FF. FFF has a larger RBE_DSB_ than FF based on the yields of DNA-DSBs as the distance to out-of-field increased.

**Conclusions:**

The out-of-field radiobiological effect of FFF could thus be greater than that of FF since the spreading of the radiation dose out-of-field with FFF could be a concern compared to the FF.

**Advances in knowledge:**

The RBE_DSB_ of FFF of out-of-field might be larger than FF.

## Introduction

Compared to the conventional flattening-filter photon beam (FF), a flattening filter-free photon beam (FFF) allows for an approximately two- to fourfold increase in the dose rate.^1–3^ The high dose rate afforded by FFF photon beams is an advantage in that it shortens the beam-on time by approx. 10–30%.^2,3^ The use of an FFF also helps minimize the intrafraction motion of tumors,^4^ which is especially relevant for cases in which a large dose per fraction is used, such as in stereotactic body radiation therapy (SBRT)^5–7^ since the dose rate of an FFF is higher than the dose rate that can be provided by an FF. Moreover, FFFs have been used for intensity-modulated radiation therapy (IMRT) and volumetric-modulated arc therapy (VMAT).^5^ Several research groups have reported a physical evaluation and dose distribution of FFF^8–10^; however, these studies were conducted with a focus other than the dose rate when the differences between FF and FFF were evaluated. The in-field and out-of-field dose profiles of the FFF were differed from those of the FF when the actual measurements and a Monte-Carlo simulation were used to evaluate.^11,12^

An FFF was irradiated with a high-dose rate photon beam,^2,3^ but there have been few radiobiological evaluations to determine the effects of dose rate variations on tumor cells. The radiobiological effects of using a cell survival fraction and an FFF were reported^13,14^; no significant difference was observed in the cell survival fraction after irradiation with a photon beam at high dose rates.^13,14^ Our 2018 study of the radiobiological effects of FFF beam using A549 (non-small-cell lung cancer) cells showed no significant difference in cell motility or the cell survival fraction.^15^ However, these studies evaluated the radiobiological effect only on the in-field dose distribution, and the effects on tumor cells based on the difference in dose profiles with the out-of-field distribution have not been established. The dose spreading out-of-field of the FFF also differed from that of the FF since an FF and an FFF have different dose profiles. It is thus necessary to evaluate the radiobiological effects of FFF in an out-of-field condition.

DNA strand breaks such as single- and double-strand breaks (SSBs and DSBs) are induced along ionizing radiation tracks in cells composed mainly of liquid water.^16,17^ Monte-Carlo simulations have been very useful to estimate the yields of DNA strand break by assessing the track structures for electrons in liquid water. Researcher have sought to conduct track structure simulations at the nanometer scale of secondary electrons and to predict the track structure of electrons for calculations of the spatial distribution of DNA hits. However, calculating both the energy deposition and the free radical reaction to DNA takes a significant amount of time to evaluate. A particle and heavy ion transport code system (PHITS) was shown to be able to simulate the track structure of electrons in liquid water in the incident energy range from 1 meV to 1 MeV,^18^ and PHITS v.3.20 could estimate the evaluation of the impacts by low energy electrons on the yields of DNA strand break induction based on the physical processes of electrons exhibited.^19^ Considering the DNA strand breaks, not only the physical conditions but also the radiobiological effectiveness was evaluated between an FF and an FFF.

In this study, we calculated the yields of DNA strand breaks (SSBs, DSBs, and the DSB/SSB ratio) to evaluate the radiobiological effectiveness of an FF and an FFF based on the difference between the in-field and out-of-field dose distributions.

## Methods and materials

Monte-Carlo simulations are calculated by the PHITS

The PHITS uses Monte-Carlo simulations code and can deal with photons, electrons, positrons, neutrons, and heavy ions.^18,20–22^ In the present study, we used the PHITS v.3.20 with the default setting, and we used the International Atomic Energy Agency (IAEA) phase-space file of the Varian TrueBeam linear accelerator (Varian Medical Systems, Palo Alto, CA).

**Figure 1.**
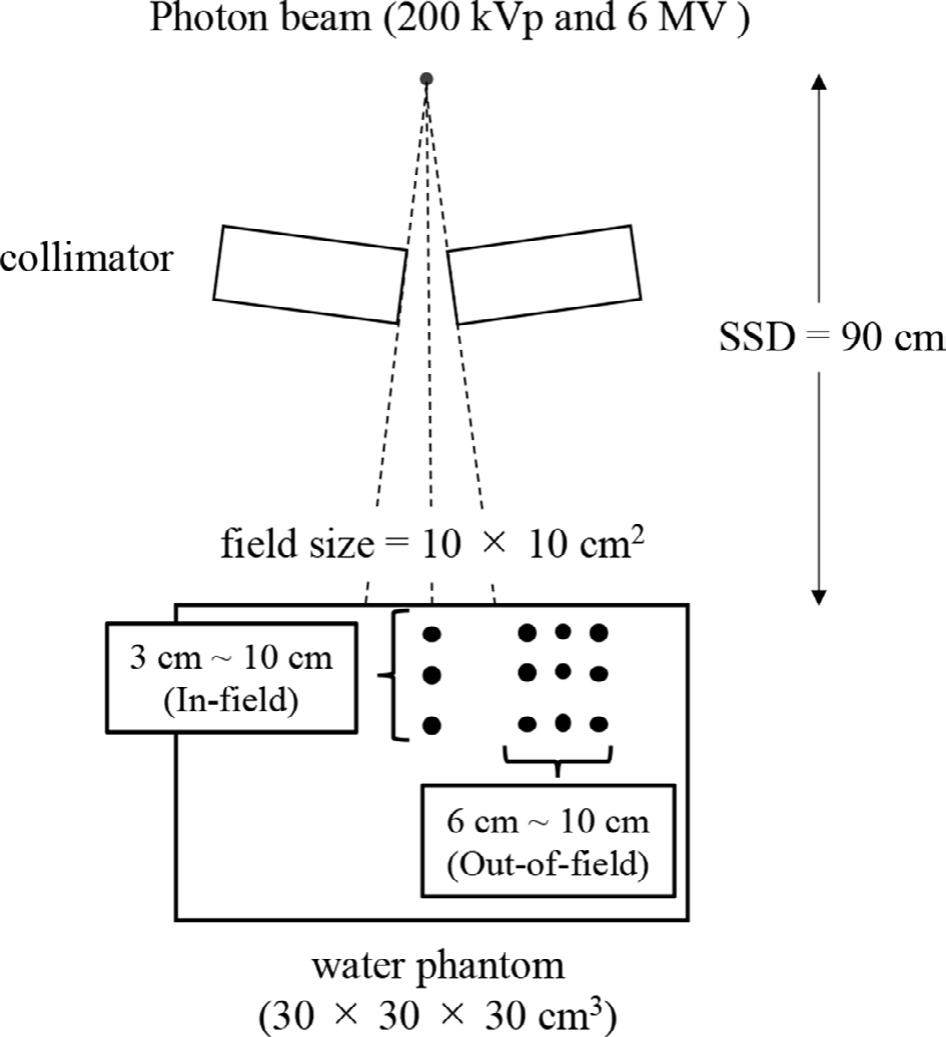
The geometries of the Monte-Carlo calculation with 220 kVp X-rays and 6 MV (FF and FFF) photon beams. The measurement points of in-field were 3-, 5-, and 10-cm depth from the surface; those of out-of-field were 6, 8, and 10 cm in the lateral direction of each depth from the center axis.

The BEAMnrc code was based on the EGSnrc platform and is optimized for modeling the treatment head of radiotherapy linear accelerators.^23^ This code includes several geometry and source subroutines, along with the variance reduction techniques to enhance simulation efficiency.^24^ The below phase-space files were made using BEAMnrc, which is built on the EGSnrc platform. These phase-space files created by BEAMnrc were transferred to the PHITS for the calculation of the dose distribution. The irradiation geometry for the 6 MV FF and FFF was as follows: a 30 × 30 × 30 cm^3^ water equivalent phantom was used; the source to skin distance (SSD) was 90 cm, and the field size was 10 × 10 cm^2^ (Figure 1). The 220 kVp photon beam, which was the reference for deriving the relative biological effectiveness (RBE), was calculated with the same geometry as that used for the 6 MV FF and FFF photon beams. The measurement points to calculate the dose-mean lineal energy and the in-field radiobiological effectiveness were located at a depth of 3, 5, and 10 cm in the water equivalent phantom on the central axis. In addition, 6, 8, and 10 cm in the lateral direction of each depth from the central axis were set as measurement points to calculate the out-of-field radiobiological effectiveness.

Calculation of dose-mean lineal energy y_D_

We calculated the physical qualities of the dose-mean lineal energy y_D_ (keV/µm) of the 6 MV FF and FFF photon beams on each measurement point in-field and out-of-field (cut-off energy = 1.0 keV). The lineal energy y in keV/μm was given by Equation (1):

(1) $$y=\frac{\varepsilon }{l}$$

The dose-mean lineal energy *y*_D_^25–27^ was calculated as:

(2) $$y_{D}=\frac{\int y^{2}f(y)dy}{\int yf(y)dy}=\frac{\int yd(y)dy}{\int d(y)dy}$$

where *ε* is the energy deposited in a domain, *l* is the mean chord length, *y* is the lineal energy, *f*(*y*) is the probability density of the lineal energy, and *d*(*y*) is the dose distribution of the lineal energy. The dose-mean lineal energy *y*_*D*_ was calculated according to ICRU report 36.^28^

Estimation of SSB, DSB, and the DSB/SSB ratio by 6 MV FF and FFF irradiation using PHITS.

PHITS v.3.20 has been shown to effectively consider the track structure code to calculate the precise electron features on a scale from a single track to DNA.^19^ The yields of strand breaks (SSBs and DSBs) using the PHITS calculation have been compared and verified with other published data including ICRU reports, the results of other simulation codes, and experimental data,^19^ and good agreement of the DSB yield calculated by PHITS with experimental DSB data has been shown for both electron and photon irradiations. By analyzing the spatial coordinates of ionization and excitation, it was possible to calculate the generation rate of DNA damage induced on charged particle tracks. The number of events per track and the number of a pair composed of two events within 3.4 nm (10 base pairs, bp) were stochastically sampled for calculating the yields of SSBs, DSBs, and the DSB/SSB ratio since the DNA strand breaks were induced by ionization and electronic excitation.^29,30^ PHITS calculates the number of SSB and then calculates the fraction of them that lie within 10 bp on opposite strands to obtain the numbers of initial DSB.

The number of events per keV *N*_event_/*E*_in_ and the number of linkages per keV *N*_link_/*E*_in_ were calculated to obtain the induction yield of SSBs and DSBs, respectively [19]. PHITS defined *k*_SSB_ and *k*_DSB_ as proportion coefficients for SSB and DSB inductions (keV/Gy/Da), respectively, *that is,* the coefficients *k*_SSB_ = 5.66 × 10^−12^ (keV/Gy/Da) and *k*_DSB_ = 1.61 × 10^−13^ (keV/Gy/Da).^19^ The numbers of SSB *Y*_SSB_ and DSB *Y*_DSB_ (/Gy/Da) as a function of electron incident energy are defined as:

(3) $$y_{SSB}(E_{in})=k_{SSB}\frac{N_{event}}{E_{in}}$$

(4) $$y_{DSB}(E_{in})=k_{DSB}\frac{N_{link}}{E_{in}}$$

We calculated the DSB/SSB ratio by using the yield ratio of DSBs and SSBs (*Y*_DSB_/*Y*_SSB_). The energy spectra of electrons by FF and FFF on each measurement point were used to calculate the yields of SSBs and DSBs and the DSB/SSB ratio.

The RBE based on the yields of DNA-DSBs

The yield of DNA-DSBs is considered an endpoint for evaluations of radiobiological effectiveness.^31,32^ The RBE obtained using the ratio of DNA-DSBs (RBE_DSB_) has been calculated for standard radiation such as 220 kVp X-rays, since the absorbed dose is related to the number of DNA-DSBs per nucleus.^33^ In the present study, the RBE_DSB_ was calculated by the ratio of the number of DNA-DSBs per nucleus with a 1.0 Gy absorbed dose when FF and FFF photon beams were irradiated on each measurement point to the number of DNA-DSBs of 220 kVp X-rays as follows:

(5) $$RBE_{DSB}=\frac{DSB_{FForFFF}}{DSB_{220kVp\;X-rays}}$$

## Results

The dose-mean lineal energy y_D_ of FF and FFF

**Figure 2.**
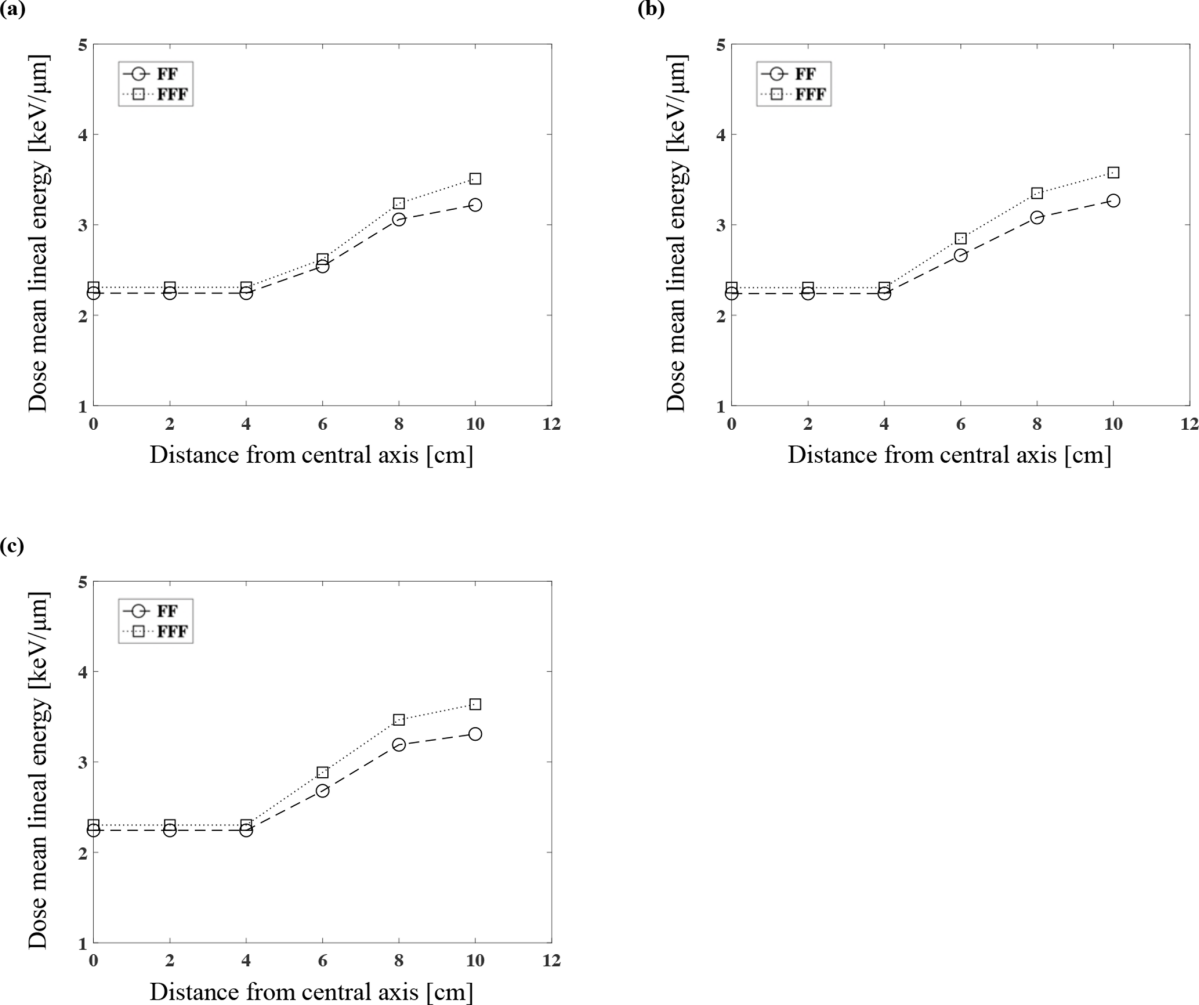
The calculations of the dose-mean lineal energy in the water equivalent phantom for the 6 MV FF and FFF at each measurement point at the depths of 3 cm (**a**), 5 cm (**b**), and 10 cm (**c**).

Our calculations of the dose-mean lineal energy y_D_ in the water equivalent phantom for the 6 MV FF and FFF are illustrated in Figure 2. The y_D_ values of the FF were 2.24, 2.54, 3.06, and 3.22 and the y_D_ values of the FFF were 2.30, 2.62, 3.24, and 3.51 (keV/µm) at each measurement point at the depth of 3 cm with 6, 8, and 10 cm in the lateral direction from the central axis, respectively (Figure 2a). At each measurement point at the depth of 5 cm, the y_D_ values of the FF were 2.24, 2.66, 3.08, and 3.27, and the y_D_ values of the FFF were 2.30, 2.84, 3.35, and 3.58 (keV/µm), respectively (Figure 2b). At each measurement point at the depth of 10 cm, the y_D_ values of the FF were 2.24, 2.68, 3.19, and 3.31 and the y_D_ values of the FFF were 2.30, 2.88, 3.47, and 3.64 (keV/µm), respectively (Figure 2c).

The FFF thus had larger y_D_ values. The difference in the water depth on the central axis (in-field) had little effect on the y_D_ values. In contrast, greater y_D_ values were observed with the greater depths and with greater lateral distances from the central axis (out-of-field).

Calculation of the DSB yield and the DSB/SSB ratios for FF and FFF by PHITS

**Figure 3.**
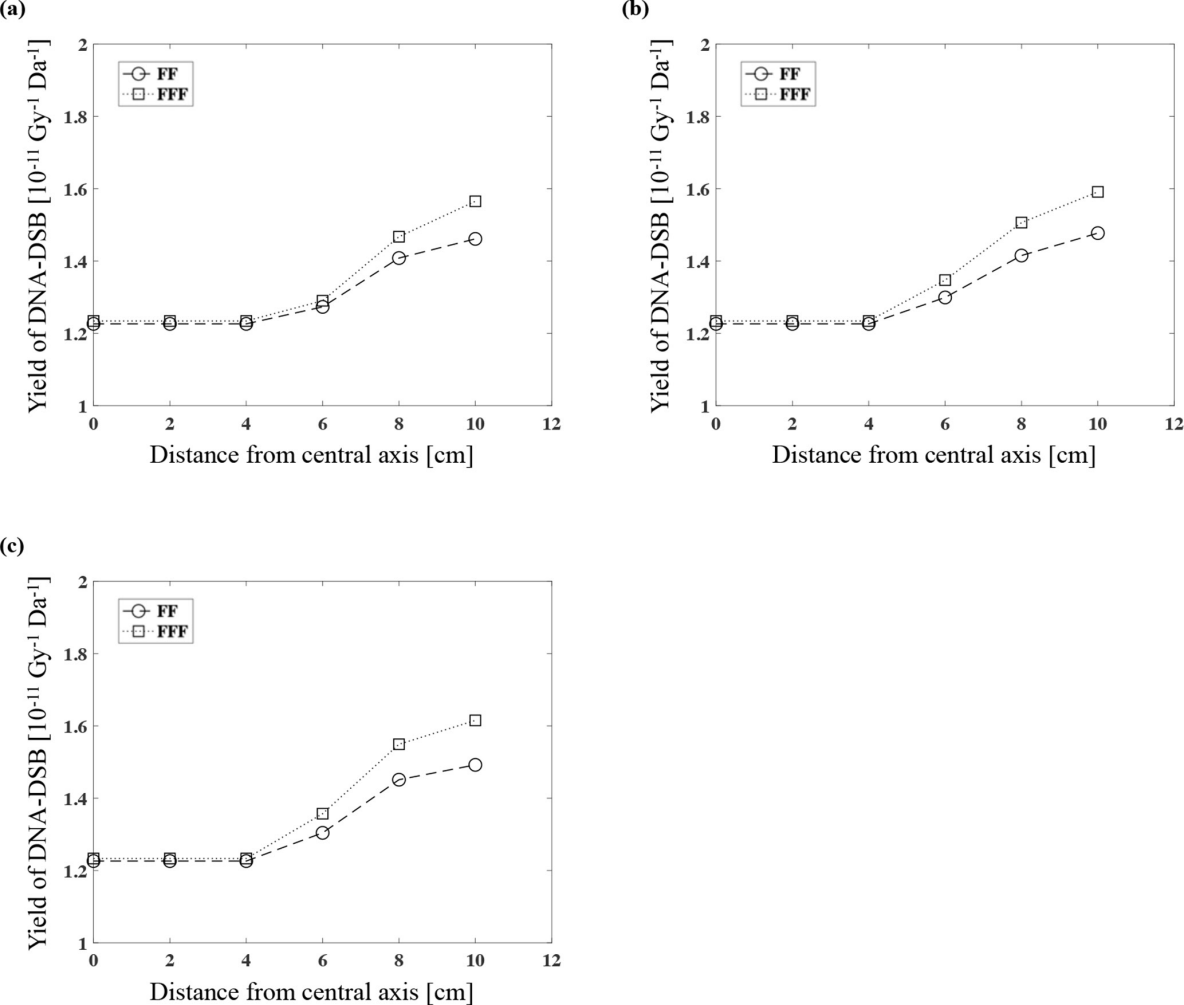
The yield of DSBs (10^−11^/Gy/Da) with 6 MV FF and FFF at each measurement point at the depth of 3 cm (**a**), 5 cm (**b**), and 10 cm (**c**).

We calculated the yield of DSBs with 6 MV FF and FFF by using the PHITS. The yields of DSBs with the FF photon beam were 1.23, 1.27, 1.41, and 1.46 and those of the FFF were 1.23, 1.29, 1.47, and 1.57 (10^−11^/Gy/Da) at each measurement point at the depth of 3 cm with 6, 8, and 10 cm in the lateral direction from the central axis, respectively (Figure 3a). At the 5 cm depth, the yields of DSBs with the FF were 1.23, 1.30, 1.42, and 1.48 and those of the FFF were 1.23, 1.35, 1.51, and 1.59 (10^−11^/Gy/Da) (Figure 3b). At the 10 cm depth, the yields of DSBs with the FF were 1.23, 1.30, 1.45, and 1.49 and those with FFF were 1.23, 1.36, 1.55, and 1.62 (10^−11^/Gy/Da), respectively (Figure 3c).

**Figure 4.**
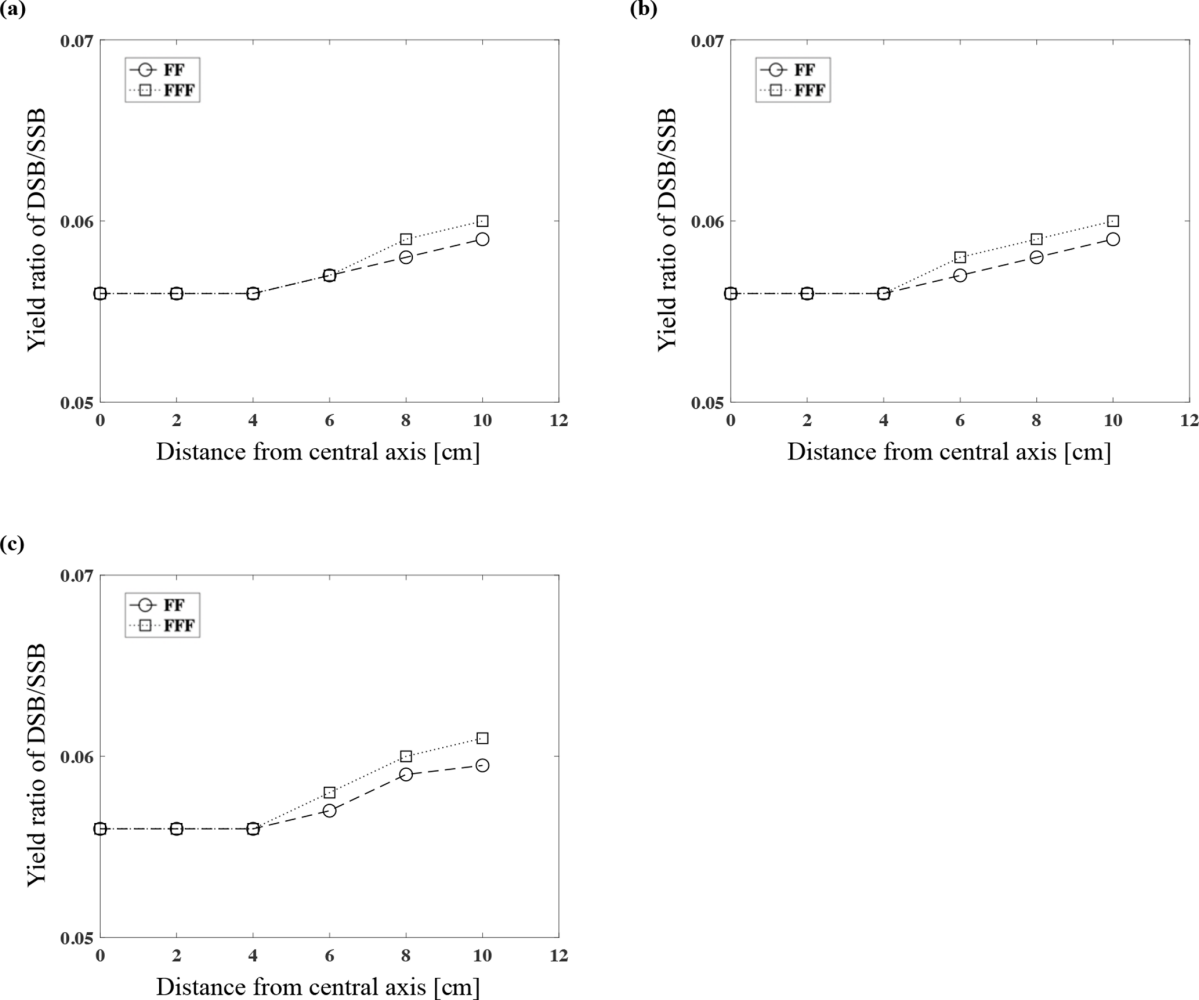
The DSB/SSB ratio for the 6 MV FF and FFF at each measurement point at the depth of 3 cm (**a**), 5 cm (**b**), and 10 cm (**c**).

We calculated the ratios of DSB/SSB with 6 MV FF and FFF. At the 3 cm depth, the ratios of DSB/SSB with the FF were 0.056, 0.057, 0.058, and 0.059, and those with the FFF were 0.056, 0.057, 0.059, and 0.060 at the three measurement points 6, 8, and 10 cm in the lateral direction from the central axis, respectively (Figure 4a). At the 5 cm depth, the ratios of DSB/SSB with the FF were 0.056, 0.057, 0.058, and 0.059, and those with the FFF were 0.056, 0.057, 0.059, and 0.060 (Figure 4b). At the 10 cm depth, the ratios of DSB/SSB with the FF were 0.056, 0.057 0.059, and 0.060, and those obtained with the FFF were 0.056, 0.058, 0.060, and 0.061 (Figure 4c). There was little effect on the yield of DSBs or the DSB/SSB ratio by changing the measured depth on the central axis in the comparison of FF and FFF (in-field), but the values were the larger with greater distance and the longer lateral distance from the central axis (out-of-field).

Relative biological effectiveness with FF and FFF based on the yields of DSBs

**Figure 5.**
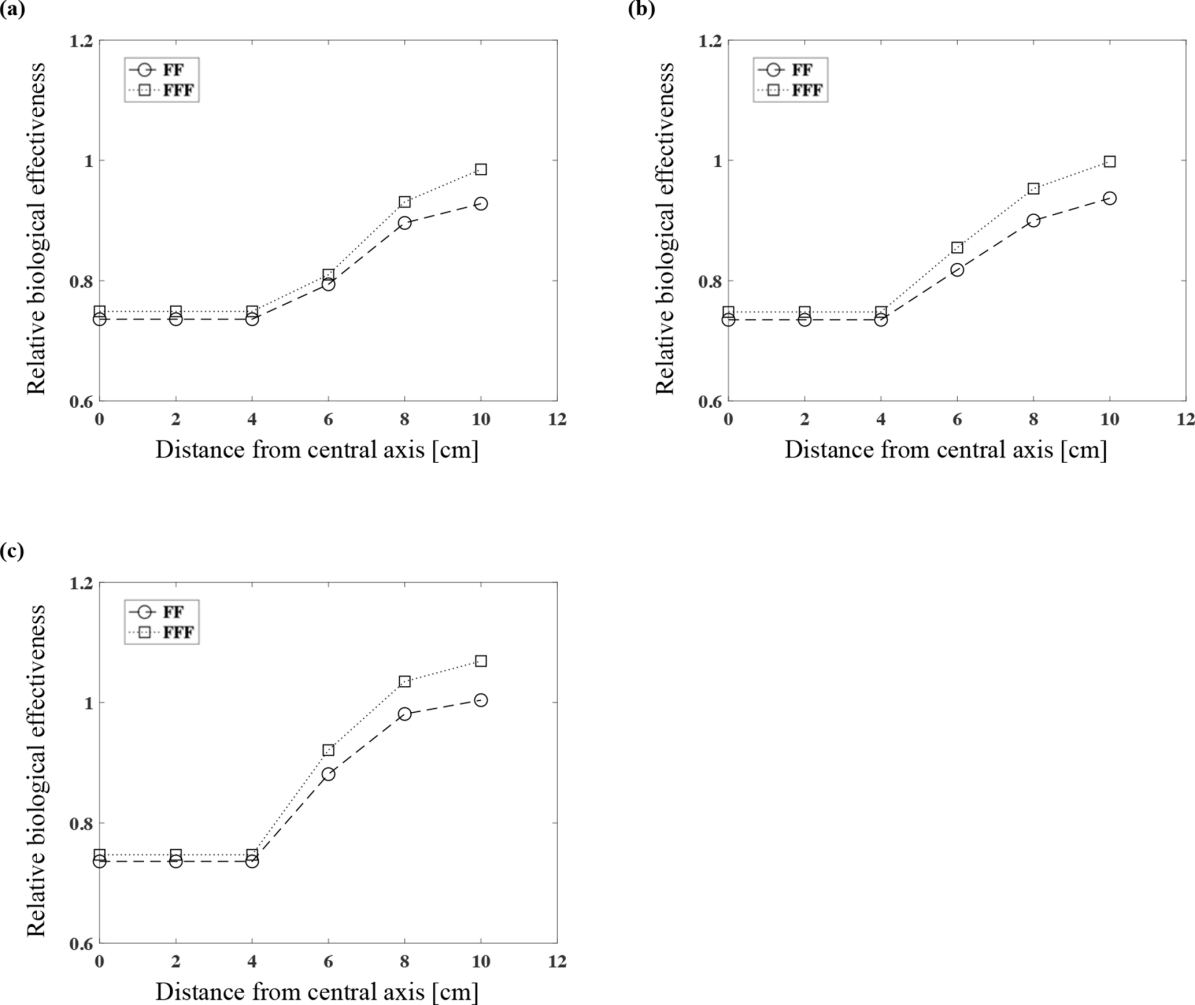
The relative biological effectiveness considering the yield of DSBs (RBE_DSB_) of 6 MV FF and FFF at each measurement point at the depth of 3 cm (**a**), 5 cm (**b**), and 10 cm (**c**).

The RBE values based on the yield of DSBs (RBE_DSB_) with 6 MV FF and FFF were calculated. At the depth of 3 cm with the measurement points at 6, 8, and 10 cm in the lateral direction from the central axis, the RBE_DSB_ values for 6 MV FF were 0.74, 0.79, 0.90, and 0.93 and those for FFF were 0.75, 0.81, 0.93, and 0.99, respectively (Figure 5a). At the 5 cm depth, the corresponding values for FF were 0.74, 0.82, 0.90, and 0.94, and those for FFF were 0.75, 0.86, 0.95, and 1.00 (Figure 5b). At 10 cm deep, the corresponding values for FF were 0.74, 0.88, 0.98, and 1.00, and those for FFF were 0.75, 0.92, 1.04, and 1.07 (Figure 5c). The RBE_DSB_ values were larger in FFF at all measurement points, and therefore FFF has a greater RBE than FF; the RBE of out-of-field was also greater.

## Discussion

After the FF was removed, the spectrum of the FFF photon beams with the Varian TrueBeam linear accelerator was softened and the proportion of low-energy components was increased compared to FF.^34^ In addition, a characteristic of the beam profile of FFF was the lower out-of-field dose distribution compared to FF.^35^ The physical qualities of the dose-mean lineal energy y_D_ values on the central axis of FF and FFF were almost the same (Figure 2). Regarding the out-of-field, the y_D_ values for FFF were larger than those for FF because FFF has more low-energy components (Figure 2).

We used the DNA-DSB yield to calculate the RBE_DSB_ values in the present study. To the best of our knowledge, this was the first study to calculate the yield of DNA-DSBs of FFF by a Monte-Carlo simulation. The RBE_DSB_ values were almost the same in-field, even when the depth changed (Figure 5). The result of in-field observation was equal to A549 cell survival comparing FF with FFF.^15^ On the other hand, the RBE_DSB_ out-of-field values increased as the depth and the distance from the central axis increased (Figure 5). We observed that compared to an FF photon beam, FFF has larger RBE_DSB_ values. We speculate that (1) the energy of the photon beam shifts to a lower energy value as the depth becomes deeper and the distance from the central axis becomes larger; and (2) the tendency is larger in FFF.

Yachi et al calculated the relationship between the calculated y_D_ values for various types of X-rays, measured the RBE_DSB_ values, and derived an approximate formula.^30^ Our present findings regarding RBE_DSB_ values are valid in comparison with the approximate formula. On the central axis (in-field), the difference of RBE_DSB_ values between FF and FFF was within 2% regardless of the depth and thus almost the same values. On the other hand, the RBE_DSB_ values of FFF were greater than those of FF as the distance to out-of-field increased. The RBE_DSB_ values of FFF were higher than those of FF by 1.7%, 4.6%, 5.6%, and 6.4% at each measurement points with a depth of 10 cm with 6 cm, 8 cm and 10 cm in the lateral direction from the central axis, respectively. The result that the RBE_DSB_ values of FFF was larger than FF was increased as the depth and the distance from the central axis increased (Figure 5). It was considered because the proportion of low dose for FFF was increased than FF with deeper and out-of-field. The increasing the proportion of low dose for FFF could also be confirmed from the results of y_D_ (Figure 2). As a result, the RBE at out-of-field of FFF might be greater than that of FF.

The advantages provided by the characteristics of FFF have enabled its use in IMRT and VMAT in clinical settings.^5^ The irradiation technique of spreading the absorbed dose out-of-field has been reported in several radiotherapy studies.^36–39^ There is also concern about a secondary cancer risk due to the spreading of the dose out-of-field.^40^ Furthermore, although IMRT and VMAT use the multi-leaf collimator (MLC) to perform intensity modulation of the dose distribution, the irradiation field was formed by only the jaw without MLC to evaluate the RBE_DSB_ value of FF and FFF in this study. The shape of the dose profile of MLC combined with jaw was different from the field by only the jaw.^41^ Therefore, it is necessary to evaluate the RBE_DSB_ value of out-of-field using the MLC. It may thus be advisable to pay attention to the spreading of the dose out-of-field when FFF is used for IMRT and VMAT.

There is a study limitation to address; we obtained the result showing that the RBE_DSB_ value of FFF was higher by approximately 6% compared to FF at the out-of-field. The clinical impact of the difference in RBE_DSB_ values between FF and FFF is not yet known. Further studies are necessary to determine the precise effects of radiobiology.

## Conclusions

FFF has greater RBE_DSB_ values than FF based on the yield of DNA-DSBs. The RBE_DSB_ values of FFF were higher than those of FF as the distance to out-of-field increased. The out-of-field radiobiological effectiveness of FFF could thus be greater than that of FF since the spreading of the radiation dose out-of-field with FFF could be a concern compared to the FF.

## References

[1] Prendergast BM, Fiveash JB, Popple RA, Clark GM, Thomas EM, Minnich DJ, et al. Flattening filter-free linac improves treatment delivery efficiency in stereotactic body radiation therapy. J Appl Clin Med Phys 2013; 14: 64–71. doi: 10.1120/jacmp.v14i3.4126 PMC5714408 23652246

[2] Kragl G, af Wetterstedt S, Knäusl B, Lind M, McCavana P, Knöös T, et al. Dosimetric characteristics of 6 and 10MV unflattened photon beams. Radiotherapy and Oncology 2009; 93: 141–6. doi: 10.1016/j.radonc.2009.06.008 19592123

[3] Tsiamas P, Sajo E, Cifter F, Theodorou K, Kappas C, Makrigiorgos M, et al. Beam quality and dose perturbation of 6 MV flattening-filter-free linac. Phys Med 2014; 30: 47–56. doi: 10.1016/j.ejmp.2013.02.004 23517668

[4] Purdie TG, Bissonnette J-P, Franks K, Bezjak A, Payne D, Sie F, et al. Cone-Beam computed tomography for on-line image guidance of lung stereotactic radiotherapy: localization, verification, and intrafraction tumor position. Int J Radiat Oncol Biol Phys 2007; 68: 243–52. doi: 10.1016/j.ijrobp.2006.12.022 17331671

[5] Pokhrel D, Halfman M, Sanford L. FFF-VMAT for SBRT of lung lesions: improves dose coverage at tumor-lung interface compared to flattened beams. J Appl Clin Med Phys 2020; 21: 26–35. doi: 10.1002/acm2.12764 31859456 PMC6964748

[6] Navarria P, Ascolese AM, Mancosu P, Alongi F, Clerici E, Tozzi A, et al. Volumetric modulated Arc therapy with flattening filter free (FFF) beams for stereotactic body radiation therapy (SBRT) in patients with medically inoperable early stage non small cell lung cancer (NSCLC. Radiother Oncol 2013; 107: 414–8. doi: 10.1016/j.radonc.2013.04.016 23725859

[7] Prendergast BM, Dobelbower MC, Bonner JA, Popple RA, Baden CJ, Minnich DJ, et al. Stereotactic body radiation therapy (SBRT) for lung malignancies: preliminary toxicity results using a flattening filter-free linear accelerator operating at 2400 monitor units per minute. Radiat Oncol 2013; 8: 273. doi: 10.1186/1748-717X-8-273 24256563 PMC3842766

[8] Dzierma Y, Licht N, Nuesken F, Ruebe C. Beam properties and stability of a flattening-filter free 7 mV beam-an overview. Med Phys 2012; 39: 2595–602. doi: 10.1118/1.3703835 22559630

[9] Montgomery L, Evans M, Liang L, Maglieri R, Kildea J. The effect of the flattening filter on photoneutron production at 10 MV in the Varian TrueBeam linear accelerator. Med Phys 2018; 45: 4711–9. doi: 10.1002/mp.13148 30141186

[10] Zavgorodni S. Monte Carlo investigation into feasibility and dosimetry of flat flattening filter free beams. Phys Med Biol 2013; 58: 7699–713. doi: 10.1088/0031-9155/58/21/7699 24140752

[11] Fogliata A, Fleckenstein J, Schneider F, Pachoud M, Ghandour S, Krauss H, et al. Flattening filter free beams from TrueBeam and versa HD units: evaluation of the parameters for quality assurance. Med Phys 2016; 43: 205–12. doi: 10.1118/1.4938060 26745913

[12] Shimozato T, Aoyama Y, Matsunaga T, Tabushi K. Beam characterization of 10-MV photon beam from medical linear accelerator without flattening filter. J Med Phys 2017; 42: 65–71. doi: 10.4103/jmp.JMP_71_16 28706351 PMC5496272

[13] King RB, Hyland WB, Cole AJ, Butterworth KT, McMahon SJ, Redmond KM, et al. An *in vitro* study of the radiobiological effects of flattening filter free radiotherapy treatments. Phys Med Biol 2013; 58: N83–94. doi: 10.1088/0031-9155/58/5/N83 23399781

[14] Karan T, Moiseenko V, Gill B, Horwood R, Kyle A, Minchinton AI. Radiobiological effects of altering dose rate in filter-free photon beams. Phys Med Biol 2013; 58: 1075–82. doi: 10.1088/0031-9155/58/4/1075 23363688

[15] Nakano H, Minami K, Yagi M, Imaizumi H, Otani Y, Inoue S, et al. Radiobiological effects of flattening filter-free photon beams on A549 non-small-cell lung cancer cells. J Radiat Res 2018; 59: 442–5. doi: 10.1093/jrr/rry041 29850845 PMC6054216

[16] Folkard M, Prise KM, Vojnovic B, Davies S, Roper MJ, Michael BD. Measurement of DNA damage by electrons with energies between 25 and 4000 eV. Int J Radiat Biol 1993; 64: 651–8. doi: 10.1080/09553009314551891 7903332

[17] Watanabe R, Rahmanian S, Nikjoo H. Spectrum of Radiation-Induced Clustered Non-DSB Damage - A Monte Carlo Track Structure Modeling and Calculations. Radiat Res 2015; 183: 525–40. doi: 10.1667/RR13902.1 25909147

[18] Sato T, Iwamoto Y, Hashimoto S, Ogawa T, Furuta T, Abe S-ichiro, et al. Features of particle and heavy ion transport code system (PHITS) version 3.02. J Nucl Sci Technol 2018; 55: 684–90. doi: 10.1080/00223131.2017.1419890

[19] Matsuya Y, Kai T, Yoshii Y, Yachi Y, Naijo S, Date H, et al. Modeling of yield estimation for DNA strand breaks based on Monte Carlo simulations of electron track structure in liquid water. J Appl Phys 2019; 126: 124701. doi: 10.1063/1.5115519

[20] Sato T, Kase Y, Watanabe R, Niita K, Sihver L, et al. Biological dose estimation for charged-particle therapy using an improved PHITS code coupled with a microdosimetric kinetic model. Radiat Res 2009; 171: 107–17. doi: 10.1667/RR1510.1 19138056

[21] Sato T, Furusawa Y. Cell survival fraction estimation based on the probability densities of domain and cell nucleus specific energies using improved Microdosimetric kinetic models. Radiat Res 2012; 178: 341–56. doi: 10.1667/RR2842.1 22880622

[22] Sato T, Hamada N. Model assembly for estimating cell surviving fraction for both targeted and nontargeted effects based on microdosimetric probability densities. PLoS One 2014; 9: e114056. doi: 10.1371/journal.pone.0114056 25426641 PMC4245256

[23] Rogers DW, Walters B, Kawrakow I, manual Busers. National Research Council of Canada Report PIRS-0509(A) revL. Ottawa, Canada: NRCC; 2016.

[24] Rogers DW, Faddegon BA, Ding GX, Ma CM, We J, Mackie TR. Beam: a Monte Carlo code to simulate radiotherapy treatment units. Med Phys 1995; 22: 503–24. doi: 10.1118/1.597552 7643786

[25] Sato T, Kase Y, Watanabe R, Niita K, Sihver L. Biological dose estimation for charged-particle therapy using an improved PHITS code coupled with a microdosimetric kinetic model. Radiat Res 2009; 171: 107–17. doi: 10.1667/RR1510.1 19138056

[26] Sato T, Furusawa Y. Cell survival fraction estimation based on the probability densities of domain and cell nucleus specific energies using improved microdosimetric kinetic models. Radiat Res 2012; 178: 341–56. doi: 10.1667/RR2842.1 22880622

[27] Sato T, Hamada N. Model assembly for estimating cell surviving fraction for both targeted and nontargeted effects based on microdosimetric probability densities. PLoS One 2014; 9: e114056. doi: 10.1371/journal.pone.0114056 25426641 PMC4245256

[28] ICRU report 36, International Commission on radiation units and measurements (ICRU, Bethesda. MD 1983;.

[29] Date H, Yoshii Y, Sutherland KL. Nanometer site analysis of electron tracks and dose localization in bio-cells exposed to X-ray irradiation. Nuclear Instruments and Methods in Physics Research Section B: Beam Interactions with Materials and Atoms 2009; 267: 1135–8. doi: 10.1016/j.nimb.2009.02.048

[30] Yoshii Y, Sasaki K, Matsuya Y, Date H, et al. Cluster analysis for the probability of DSB site induced by electron tracks. Nuclear Instruments and Methods in Physics Research Section B: Beam Interactions with Materials and Atoms 2015; 350: 55–9. doi: 10.1016/j.nimb.2015.03.025

[31] Gerelchuluun A, Hong Z, Sun L, Suzuki K, Terunuma T, Yasuoka K, et al. Induction of in situ DNA double-strand breaks and apoptosis by 200 MeV protons and 10 mV x-rays in human tumour cell lines. Int J Radiat Biol 2011; 87: 57–70. doi: 10.3109/09553002.2010.518201 20954835

[32] Franken NAP, ten Cate R, Krawczyk PM, Stap J, Haveman J, Aten J, et al. Comparison of RBE values of high- let α-particles for the induction of DNA-DSBs, chromosome aberrations and cell reproductive death. Radiat Oncol 2011; 6: 64. doi: 10.1186/1748-717X-6-64 21651780 PMC3127784

[33] Yachi Y, Yoshii Y, Matsuya Y, Mori R, Oikawa J, Date H. Track structure study for energy dependency of electrons and x-rays on DNA double-strand break induction. Sci Rep 2019; 9: 17649. doi: 10.1038/s41598-019-54081-6 31776470 PMC6881292

[34] Cashmore J. The characterization of unflattened photon beams from a 6 MV linear accelerator. Phys Med Biol 2008; 53: 1933–46. doi: 10.1088/0031-9155/53/7/009 18364548

[35] Sangeetha S, Sureka CS. Comparison of flattening filter (FF) and Flattening-Filter-Free (FFF) 6 mV photon beam characteristics for small field dosimetry using EGSnrc Monte Carlo code. Radiation Physics and Chemistry 2017; 135: 63–75. doi: 10.1016/j.radphyschem.2017.02.029

[36] Swanson EL, Indelicato DJ, Louis D, Flampouri S, Li Z, Morris CG, et al. Comparison of three-dimensional (3D) conformal proton radiotherapy (RT), 3D conformal photon RT, and intensity-modulated RT for retroperitoneal and intra-abdominal sarcomas. Int J Radiat Oncol Biol Phys 2012; 83: 1549–57. doi: 10.1016/j.ijrobp.2011.10.014 22270176

[37] Haertl PM, Pohl F, Weidner K, Groeger C, Koelbl O, Dobler B. Treatment of left sided breast cancer for a patient with funnel chest: volumetric-modulated Arc therapy vs. 3D-CRT and intensity-modulated radiotherapy. Med Dosim 2013; 38: 1–4. doi: 10.1016/j.meddos.2012.04.003 22727550

[38] Moon SH, Shin KH, Kim TH, Yoon M, Park S, Lee D-H, et al. Dosimetric comparison of four different external beam partial breast irradiation techniques: three-dimensional conformal radiotherapy, intensity-modulated radiotherapy, helical tomotherapy, and proton beam therapy. Radiother Oncol 2009; 90: 66–73. doi: 10.1016/j.radonc.2008.09.027 18992950

[39] Beckham WA, Popescu CC, Patenaude VV, Wai ES, Olivotto IA. Is multibeam IMRT better than standard treatment for patients with left-sided breast cancer? Int J Radiat Oncol Biol Phys 2007; 69: 918–24. doi: 10.1016/j.ijrobp.2007.06.060 17889273

[40] Zhang Q, Liu J, Ao N, Yu H, Peng Y, Ou L, et al. Secondary cancer risk after radiation therapy for breast cancer with different radiotherapy techniques. Sci Rep 2020; 10: 1220. doi: 10.1038/s41598-020-58134-z 31988348 PMC6985127

[41] Pönisch F, Titt U, Vassiliev ON, Kry SF, Mohan R. Properties of unflattened photon beams shaped by a multileaf collimator. Med Phys 2006; 33: 1738–46. doi: 10.1118/1.2201149 16872081

